# Comparative analysis of anti-viral transcriptomics reveals novel effects of influenza immune antagonism

**DOI:** 10.1186/s12865-015-0107-y

**Published:** 2015-08-14

**Authors:** Juilee Thakar, Boris M. Hartmann, Nada Marjanovic, Stuart C. Sealfon, Steven H. Kleinstein

**Affiliations:** Department of Pathology, Yale School of Medicine, New Haven, CT 06510 USA; Department of Neurology, Icahn School of Medicine at Mount Sinai, New York, NY 10029 USA; Center for Translational Systems Biology, Icahn School of Medicine at Mount Sinai, New York, NY 10029, USA; Interdepartmental Program in Computational Biology and Bioinformatics, Yale University, New Haven, CT USA; Department of Microbiology and Immunology, University of Rochester, Rochester, NY 14642 USA; Department of Biostatistics and Computational Biology, University of Rochester, Rochester, NY 14642 USA

**Keywords:** Gene set enrichment analysis, Transcription factor activity, Mathematical modeling, Influenza antagonism, Dendritic cells

## Abstract

**Background:**

Comparative analysis of genome-wide expression profiles are increasingly being used to study virus-specific host interactions. In order to gain mechanistic insights, gene expression profiles can be combined with information on DNA-binding sites of transcription factors to detect transcription factor activity (by analysis of target gene sets) during viral infections. Here, we apply this approach to study mechanisms of immune antagonism elicited by Influenza A virus (New Caledonia/20/1999) by comparing the transcriptional response with the non-pathogenic Newcastle disease virus (NDV), which lacks human immune antagonism.

**Results:**

Existing gene set approaches do not quantify activity in a way that can be statistically compared between responses. We thus developed a new method for Bayesian Estimation of Transcription factor Activity (BETA) that allows for such quantification and comparative analysis across multiple responses. BETA predicted decreased ISGF3 activity during influenza A infection of human dendritic cells (reflected in lower expression of Interferon Stimulated Genes, ISGs). This prediction was confirmed through a combination of mathematical modeling and experiments at different multiplicities of infection to show that ISGs were specifically blocked in infected cells. Suppression of the transcription factor SATB1 was also predicted as a novel effect of influenza-mediated immune antagonism, and validated experimentally.

**Conclusions:**

Comparative analysis of genome-wide transcriptional profiles can reveal new effects of viral immune antagonism. We have developed a computational framework (BETA) that enables quantitative comparative analysis of transcription factor activities. This method will aid future studies to identify mechanistic differences in the host-pathogen interactions. Application of BETA to genome-wide transcriptional profiling data from human DCs identified SATB1 as a novel effect of influenza antagonism.

**Electronic supplementary material:**

The online version of this article (doi:10.1186/s12865-015-0107-y) contains supplementary material, which is available to authorized users.

## Background

Circulating strains of influenza A virus (IAV) vary year-to-year in their genetic composition and potential pathogenicity. Influenza associated deaths ranged from 3000 to 49,000 per year in the United States between 1976 and 2007 [1, 2]. The impact of IAV on the human population is influenced by the periodic occurrence of IAV pandemics, such as the one in 2009 that resulted in the deaths of more than 18,000 people worldwide [3, 4]. These pandemics are driven by the introduction of new IAV strains for which there is no (or minimal) pre-existing immunity. The 2009 IAV pandemic strain was a result of a previous triple reassortment of bird, swine and human flu viruses further combined with a Eurasian pig flu virus. The ability to predict the immune responsiveness to such emerging strains would greatly improve our ability to respond appropriately to these threats.

The IAV genome has evolved several mechanisms to antagonize host immune responses. NS1 and PB1-F2 are particularly important since they directly interact with and modulate cellular innate immune response [5–7]. For example, NS1 binds and sequesters double-stranded RNA (dsRNA) produced by the virus, thereby preventing activation of host pathogen-recognition receptors (PRRs); delaying the production of interferon beta (IFNβ). Mutations in these immune antagonists have been associated with increased symptoms in circulating IAV strains [8, 9]. Thus, understanding mechanisms of IAV antagonism is critical to be able to predict pathogenicity.

Genome-wide transcriptional profiling studies offer an unbiased approach to investigating host immune responses, and have identified several markers associated with severe IAV infections [8, 10–12]. Severe infections are generally characterized by an early, sustained and excessive inflammatory response [8, 10–12]. This inflammatory response is regulated by NFkB, HMGA1 and NFATC4 transcription factors [12]. In contrast, asymptomatic infections are associated with the induction of negative regulators of inflammatory signals, especially NLRP3 and NOD2 [11, 12]. Many transcription factors, including IRF7, STAT1 and NFkB1, are induced by all IAV strains [13, 14]. Nevertheless, comparison across multiple IAV strains has revealed strain-specific effects on the rate and magnitude of the innate immune responses [15]. Many of these strain-specific effects are likely due to differences in virally-encoded immune antagonists. For example, the 2009 pandemic strain lacks the ability to interact with the cellular pre-mRNA processing protein CPSF30 [16] and does not code for the virulence factor PB1-F2 [17]. Moreover, deletion of NS1 increases the number and magnitude of expression of cellular genes implicated in the IFN, NF-κB, and other antiviral pathways [7, 8, 18, 19]. However, reproducible signatures of IAV antagonisms are yet to be revealed [20]. Immune antagonism is frequently associated with the suppression of activity (e.g., production of interferon and interferon induced proteins, such as OAS and PKC is suppressed by non-structural (NS1) protein of IAV) [7]. Thus, detecting such mechanisms depends on selecting a good control where the activity is present. One possibility is to compare the response to multiple strains, while an alternate strategy uses a virus that is non-pathogenic in humans, such as Newcastle Disease Virus (NDV). NDV infection of humans provides an ideal system to define the uninhibited regulatory network [21, 22]. NDV is an avian virus that is able to stimulate innate immunity similar to IAV, but lacks the ability to evade the human immune response [23]. By comparing the activity of transcription factors between the IAV and NDV responses, we can gain insights into the effects of viral antagonism leading to the suppression host immunity. While the differences in transcription factors activities between the IAV and NDV responses could also be mediated by the involvement of yet unknown viral recognition pathways, the current state of knowledge indicates striking similarity in the anti-viral responses which are mainly mediated by the Rig-I signaling pathway in both infections. Moreover, at the same MOI IAV and NDV show similar infectivity in DCs [24, 25]. Identifying active pathways and transcription factors [13], and then comparing these between multiple responses could be used to identify the effect of IAV immune antagonism.

Several computational methods have been proposed to detect transcription factor activity from genome-wide transcriptional profiling data [13, 26]. These methods are based on the analysis of coordinated changes in the expression of transcription factor target genes. These include over-representation approach such as hypergeometric test, or aggregate score approach such as gene set enrichment analysis (GSEA) [27]. These methods can be used to identify transcription factors with significant activity in one response that is lacking in another response [28]. In addition to being sensitive to the cutoffs used for statistical analysis, this approach is subject to Simpson’s paradox [29]. Just because two responses behave differently from their respective controls does not imply that they are significantly different from each other. For example, just because a gene is significantly differentially-expressed following NDV infection (vs. control), but not IAV infection (vs. control), does not necessarily mean the gene will be differentially expressed when comparing NDV and IAV directly. Furthermore, the p-values produced by such methods cannot be directly compared between two infections because lower p-values do not necessarily imply stronger activity. Thus, more flexible methods are needed that allow for quantification of transcription factor and pathway activities and their direct comparison across studies.

We propose a new approach for Bayesian Estimation of Transcription Factor Activity (BETA) that quantifies activity (rather than simply detecting it), and allows for direct comparison between multiple responses. Applying BETA to compare the transcription factor activity of human dendritic cells to infection with the New Caledonia strain of IAV with the non-pathogenic NDV response reveals several effects of IAV-mediated antagonism on transcription factors, including a novel effect on SATB1, which we have validated experimentally.

## Results

### BETA enables comparative analysis across time-points and virus strains

To compare the IAV and NDV responses, transcription factor activity (A) was defined independently for each infection response as the log-odds ratio between the observed frequency of TF targets among differentially-expressed genes (*π*) compared with the expected TF target frequency among a set of background genes ($\widehat{\pi}$):

$$ A\equiv { \log}_{10}\frac{\pi /\left(1-\pi \right)}{\widehat{\pi}/\left(1-\widehat{\pi}\right)} $$

Thus, positive values of A indicate that TF targets are more frequent among differentially-expressed genes than expected (i.e., the TF is actively inducing its target-genes), while negative values indicate they are less frequent (i.e., the TF is actively suppressing its target-genes). By analyzing the behavior of target genes, this approach does not require that the TF gene itself be differentially expressed. The use of log-odds ratio allows the activity (A) to be compared between IAV and NDV responses, and more generally between experimental condition and time-points. In addition, the observed frequency of TF targets (*π*) and the activity A are characterized by a full probability density function (PDF) that is estimated using a Bayesian approach as described in Additional file 1: Text S1 and [30]. The Bayesian Estimation of Transcription factor Activity (BETA) allows for activities to be compared across time-points, microarray platforms and experiments since it accounts for differences in both the number of differentially-expressed genes (e.g., from differing experimental quality), as well as the background frequency of TF targets (e.g., because different microarrays can measure different sets of genes).

**Fig. 1:**
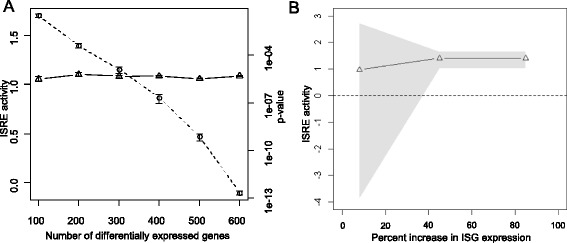
BETA is robust to the number of differentially-expressed genes, but sensitive to gene expression changes: (**a**) Comparison of transcriptional profiles from DCs stimulated with Interferon-β for 2.5 h and unstimulated DCs identified 884 differentially-expressed genes. Varying numbers of genes (x-axis) were randomly sampled from this set. For each of these cases, ISGF3 activity was measured by BETA (left y-axis and empty triangles) and hypergeometric test P values (right y-axis and empty circles) (**b**) Starting from the transcriptional profile of un-stimulated DCs, ISGF3 activity was simulated by computationally increasing the expression levels of ISGs, defined by Schoggins et. al. [31], from 8 to 85 % above their baseline values (x-axis). ISGF3 activity was then measured by BETA (y-axis, with grey area indicating 95 % confidence intervals) using a gene set based on the V$ISRE_01 matrix from TRANSFAC. P-values ISGF3 activity are 3.00e-1, 2.12e-13 and 1.07e-13

To confirm that the activity estimated by BETA is robust to the number of differentially expressed genes, we quantified ISGF3 activity in human dendritic cells stimulated with IFN-β. ISGF3 (a complex of STAT1, STAT2 and IRF9) is activated by IFN-β and leads to the up-regulation of several hundred interferon stimulated genes (ISGs) [31]. IFN-β stimulations of DCs for 2.5 h induce 884 genes. To quantify ISGF3 activity when the number of DE genes vary, different numbers of differentially expressed genes were sampled from these 884 and the set of TF targets was determined among them by computationally defining the genes having TRANSFAC binding site matrix for ISGF3 (V$ISRE_01) in the promoter region (see Materials and methods). While the P-values produced by the hypergeometric test decrease significantly as the number of differentially-expressed genes increases, BETA activity remained constant (Fig. 1a). As a positive control, ISGF3 activity was simulated as the increased expression of ISGs (defined by Schoggins et. al. [31]) with 8 to 85 % of their pre-stimulation levels (Fig. 1b). Fig. 1b shows that the activity estimated by BETA significantly increases and the 95 % confidence intervals become tighter as ISG expression levels increase. Thus, BETA activities are sensitive to underlying gene expression differences and can be used to compare experiments with differing numbers of differentially-expressed genes (e.g., because of altered variance or differences in the number of genes being measured).

### Transcription factors antagonized by IAV during the early response (≤6 hpi)

**Fig. 2:**
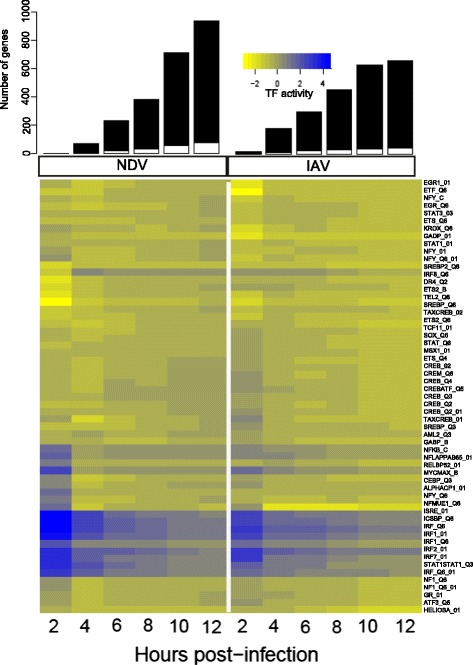
Transcription factor activities following IAV and NDV infections. The number of up-regulated genes was determined by comparing expression at each time-point post-infection with pre-infection levels (upper bar plots). Up-regulated TFs are indicated by the white portion of each bar. BETA was then used to quantify TF activity (lower heatmap) in NDV and IAV infections based on gene sets defined by TRANSFAC matrices (rows). Coloring indicates weak (yellow) to strong (blue) TF activity

**Fig. 3:**
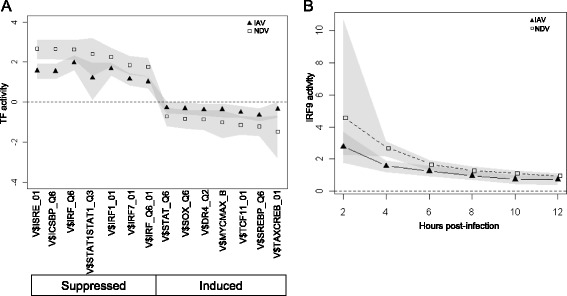
Transcription factors suppressed by IAV within 6 h post-infection. **a** BETA was used to identify transcription factor matrices with significantly different activity within 6 h following IAV infection (triangles) compared to NDV infection (squares) (q < 0.1). Only TFs that are also differentially-expressed at the mRNA level are shown. **b** ISGF3 activity (y-axis) was calculated by BETA using the V$ISRE_01 matrix following IAV infection (triangles) and NDV infection (squares). The grey area indicates the 95 % confidence interval for activity, with the dashed line representing the absence of transcriptional activity

To detect the effects of IAV antagonism on host immunity, TF activities were compared between the responses to IAV and NDV. Since NDV infection activates innate viral sensing pathways, but does not encode any known human immune antagonists, any differences between these responses suggest modulation of the immune response by IAV. As a first step, BETA was used to define the set of TFs with significant activity in either infection (IAV or NDV). The statistical significance of the BETA activity from zero was determined using Equation 7 in [30]. TF target gene sets were created based on the presence of a TRANSFAC-defined binding site in the promoter region (see Materials and methods). When considering genes up-regulated during the first 6 hpi (Fig. 2, upper panel), significant BETA activity was found for 101 TFs, with 11 of these TFs also differentially-expressed at the mRNA level (Fig. 2, and Additional file 1: Text S2). However, many of the differentially expressed TFs were down-regulated with minimal changes over time, in contrast to the up-regulated TFs, which showed significant changes in their activities. Strong activity was detected for many factors with well-characterized involvement in antiviral responses, including of ISGF3, NFKB1, CREB and STAT-family TFs [13, 14]. All of these factors displayed significant activity following both IAV and NDV infections. In fact, when using BETA activity and mRNA differential-expression as criteria, no TFs were found to be specific for the IAV or NDV responses. However, taking advantage of the ability to quantitatively compare BETA activities across responses rather than simple presence/absence, several of these TFs were found to have significantly different levels of activity between the two infections (Fig. 3a). In each case, the absolute activity following IAV infection was suppressed. Seven TF target gene sets with positive activity in both responses had significantly lower activity following IAV infection. All of these sets were defined by IRF/ISRE binding sites, which have highly related motifs that are difficult to differentiate. The remaining seven TF target gene sets, including SOX10 and NFE2L1 targets, had negative activity and were less suppressed following IAV infection. Negative BETA activity could indicate that these TFs have repressive roles as a normal part of the immune response. However, none were found to have significant activity among down-regulated genes, and they were not investigated further. Several of the active TFs predicted by BETA were also identified using other existing methods (hypergeometric test, GSEA and QuSAGE), but a comparative analysis indicated that BETA has better power (Additional file 1: Text S3). Overall, these results suggest that the interferon response is a target of IAV immune antagonism for the NC strain of IAV, as has previously been shown for other strains [14]. This antagonism was strongest in the early phase of the response, but could be detected at later time-points as well (Fig. 3b).

### Interferon stimulated genes are specifically suppressed in IAV-infected cells

Significant ISGF3 activity was observed during both IAV and NDV responses (P < 0.05). However, quantitative differences were apparent when comparing the strength of this activity between the two viruses. This raises an important question: if IAV blocks the interferon response (as BETA predicts), why are so many ISGs, such as MX1, still differentially-expressed? To explain this observation, we hypothesized that IAV was indeed blocking ISG expression in infected cells, but that ISGs were still being expressed in non-infected cells. Since the DCs were infected with IAV at an MOI of 1, it is expected that approximately 63 % of cells would not be infected. This hypothesis predicts that the lower ISGF3 activity observed following IAV infection results from significant heterogeneity in the underlying population being measured (with the microarray data reflecting the population average).

**Fig. 4:**
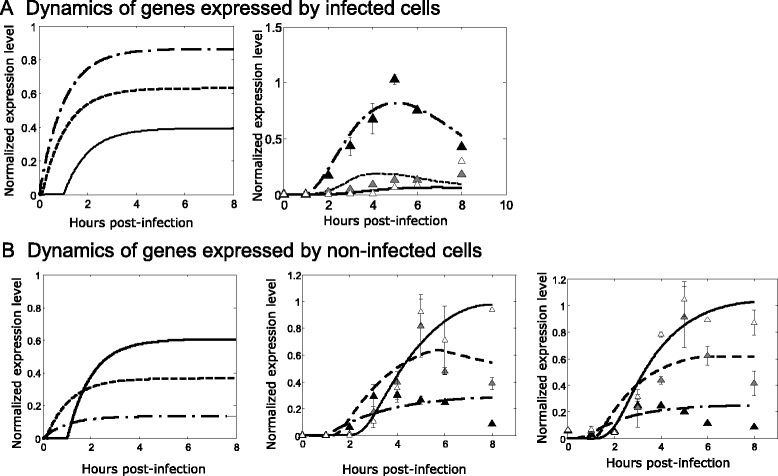
Model-based analysis of genes at different multiplicities of infection (MOI). **a** An ODE model (equation 1) was used to predict the dynamics of genes expressed by infected cells (left plot) at different MOIs (individual lines). The same model was then fit to experimentally measured IFNA expression profiles (right plot) at different MOIs. Best fit parameter values were (*k*_*i*_ = 1.6, *n* = 1.14, *d* = 0.62 and *H* = 0.99). **b** An ODE model (equation 2) was used to predict the dynamics of genes expressed by non-infected cells (left plot) at different MOIs. The same model was then fit to experimentally measured MX1 (middle plot) and IP10 (right plot) expression profiles. Best fit parameter values for MX1 were *k*_*i*_ = 1.4, *n* = 4.1, *d* = 0.73 and *H* = 0.07, and for IP10 were *k*_*i*_ = 1.18, *n* = 4.4, *d* = 0.56 and *H* = 0.08. In all plots, lines indicate model results and triangles show experimentally measured values for MOI = 0.5 (empty triangles and continuous lines), MOI = 1 (grey triangles and dashed lines) and MOI = 2 (black triangles and dash-dot lines)

To better understand how the observed gene expression patterns could be affected by underlying population heterogeneity, we developed a dynamic model of ISG induction. In this model, gene transcription increases as a function of the amount of interferon (due to autocrine and paracrine signaling), and interferon levels are approximated by the observed expression of IFNB1 mRNA (see Materials and methods for details). Three extreme cases were considered: (1) a gene was expressed by both infected and non-infected cells, (2) a gene was expressed only by infected cells, and (3) a gene was expressed only by non-infected cells. In each of these cases, we simulated gene expression over 8 h and following infection with MOI = 0.5, 1 and 2, corresponding to 39, 63 and 86 % of cells being infected based on the Poisson distribution, respectively. For genes that are expressed by all cells (case 1), the model predicts that the temporal expression profile will be independent of the MOI. For genes that are expressed only by infected cells (case 2), the model predicts that increasing MOI will lead to higher expression levels, but later induction times (Fig. 4a). Finally, for genes that are expressed only by non-infected cells (case 3), the model predicts that increasing MOI will lead to earlier and lower expression levels (Fig. 4b). The parameter estimation protocol (see Materials and methods section) was evaluated by recalling the parameter values in the simulated data. We found that recall of all parameters was good as estimated by normalized root mean squared error (NRMSE) (NRMSE values are 0.12 for ki/ku/kb, 0.18 for n, 0.05 for d and 0.14 for k) (Additional file 2: Figure S2). Thus, qualitatively different behaviors were predicted depending on which type(s) of cells are expressing the gene.

To test the prediction that the ISG expression observed following IAV infection was predominantly coming from non-infected cells, DCs were infected with IAV at three multiplicities of infections (MOIs): 0.5, 1 and 2. Consistent with expectations based on the Poisson distribution, the number of infected cells was 25, 60 and 83 % for MOIs of 0.5, 1 and 2, respectively. Gene expression was measured for IFNA and two ISGs (MX1 and IP10). The expression pattern of IFNA served as a positive control, since it should be expressed only by infected cells. As predicted by the model (case 2), peak IFNA levels were positively correlated with MOI, but negatively correlated with the time of initial induction (Fig. 4a right panel). In contrast, MX1 and IP10 expression decreased with increasing MOI. This behavior is consistent with expression from non-infected cells (case 3) and indicates that infected cells contribute little, if anything, to the observed expression profiles. Moreover, IP10 was expressed earlier at higher MOIs, suggesting that higher number of infected cells drive earlier expression of some ISGs. This time of activation of IP10 was more sensitive to the number of infected cells than MX1. Such sensitivity to the number of infected cells could arise from differential bindingof IRF9, a hypothesis which is not further explored here. Thus, ISGs were expressed mainly by non-infected cells following IAV infection, consistent with the hypothesis that IAV antagonizes the type I interferon response specifically in infected cells.

### SATB1 is antagonized by IAV

**Fig. 5:**
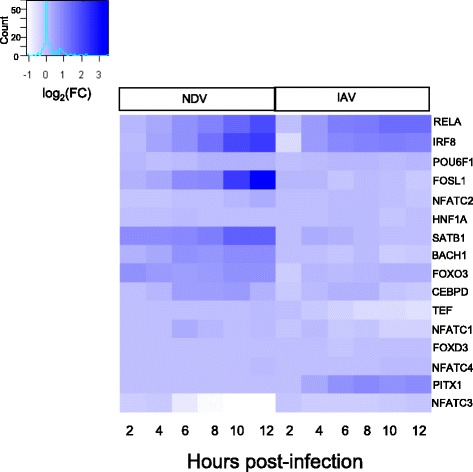
mRNA expression profiles for transcription factors modulated by IAV at late time points (>6 hpi). Log_2_ fold changes across time (columns) for transcription factors (rows) with significantly different BETA activities (q < 0.05) in IAV infection compared to NDV infection. Coloring indicates small (white) to large (blue) fold changes with respect to unstimulated expression levels

**Fig. 6:**
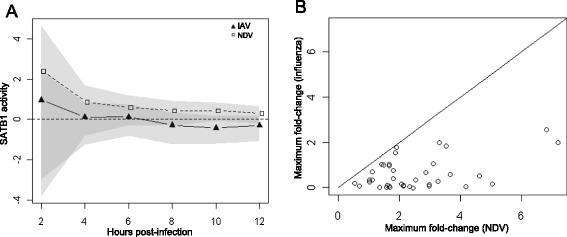
Prediction of SATB1 as a target of IAV antagonism. **a** BETA was used to quantify the activity of SATB1 (V$SATB1_01 matrix) following infection with IAV (solid triangles) and NDV (open squares). Grey areas indicate 95 % confidence intervals for activity with overlapping intervals shown in a darker shade. The horizontal dashed line at 0 represents the absence of transcriptional activity. **b** The maximum log_2_ fold change over 12 h post-infection was determined for all SATB1 target genes (points) following IAV (y-axis) and NDV infections (x-axis). The solid line indicates equal fold changes

**Fig. 7:**
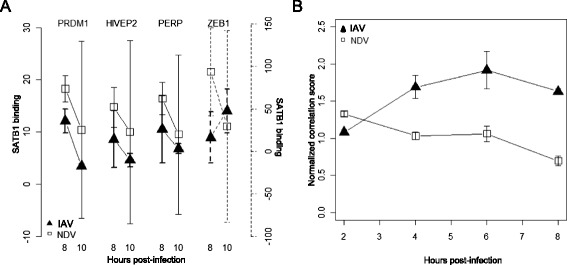
SATB1 binding and translocation are differentially modulated by IAV infection. **a** ChIP-PCR was used to measure SATB1 binding to four predicted target genes (PRDM1, HVEP2, PERP and ZEB1) at 8 and 10 h after infection with IAV (solid triangles) and NDV (open squares). **b** SATB1 nuclear translocation following infection with IAV (black solid triangles) and NDV (open squares)

To identify additional effects of IAV antagonism, BETA was used to compare TF activities between IAV and NDV infection during the later response (6-10 h post-infection). Factors identified during this time period could be involved in dendritic cell maturation and interactions with T cells. This analysis identified 16 TFs, including FOXO3 and NFAT, as potential targets of IAV antagonism (Fig. 5). Two of these TFs (SATB1 and FOSL1) also showed significantly lower mRNA expression levels following IAV infection compared with NDV infection (Fig. 5). SATB1 was particularly interesting because of its known involvement in T cell development [32–35]. BETA predicted that SATB1 activity was significantly suppressed at 10 h post-infection with IAV (Fig. 6a). This suppression impacted the maximum fold-changes achieved by virtually all SATB1 target genes (Fig. 6b). To validate the prediction that IAV infection antagonizes SATB1 activity, ChIP-PCR was carried out to quantify SATB1 binding at 8 and 10 h post-infection with IAV or NDV. Four SATB1 targets were chosen based on the computationally-predicted presence of a SATB1 binding site in the promoter region and significant differential-expression when comparing the NDV and IAV responses. In all four cases, lower binding of SATB1 was observed following IAV infection compared to NDV infection (Fig. 7a). Significant differences in SATB1 nuclear translocation were also observed (Fig. 7b). Interestingly, these measurements suggested higher levels of SATB1 in the nucleus following IAV infection. This could be a result of a feedback regulation mechanism induced by failure to induce SATB1 target genes. Taken together, these results suggest that IAV inhibits SATB1 activity, and that this inhibition is mediated through blocking of SATB1 binding to the promoters of its target genes.

## Discussion

Differences in genome-wide expression patterns are frequently used to compare virus-host interactions. Comparisons across a range hosts have revealed immune pathways involved in the detection and destruction of viruses, while comparisons across viruses have elucidated a diverse array of mechanisms used by viruses to antagonize host immunity. Mechanisms of viral antagonism include modulating antigen presentation, apoptosis of infected cells, cytokine-mediated signaling, and Fc dependent immune activation [20]. Influenza is a fast evolving virus, and has developed multiple ways of antagonizing anti-viral responses [20]. For example, IAV virus protein PB1-F2 suppresses the activation of IL-6 and IL-1β through the modulation of NFkB [36, 37]. The function of these antagonists can vary between IAV strains, and impacts pathogenicity [38, 39]. Previous work has compared wild-type IAV strains with genetically altered versions where potential antagonists, such as NS1, are knocked out or genetically altered [19]. While such comparisons can identify mechanisms directly related to individual antagonists, they do not provide a global view of virus-host interaction and some mechanisms may be masked through redundancy. Here, we propose to use NDV, a non-pathogenic virus, as a control to identify the genome-wide effects of IAV immune antagonism. Influenza and NDV are both negative sense RNA myxoviruses and in birds, which are their natural hosts, their clinical symptoms are similar. NDV and IAV both activate anti-viral responses mediated by Rig-I and IPS-1 [40]. However, NDV does not encode any known human immune antagonists, and thus can be used as a baseline to identify pathways and transcription factors whose activity is “missing” during IAV infections.

Viruses can modulate host responses through multiple mechanisms [20]. In order to gain mechanistic insight, we sought to use differential regulatory patterns of genome-wide expression profiles to identify candidate transcriptional activity differences. Similar to many previous studies, transcription factor activity was assessed by analyzing the behavior of putative target genes [26]. Existing methods, such as GSEA [27] are focused on detecting activity, and their main output consists of P-values indicating whether the factor is active. However, focusing on P-values limits the ability to carry out post-hoc comparative studies, since lower P-values do not necessarily imply higher activity. BETA enables easy comparison across experiments and time-points by quantifying transcription factor activity rather than simply detecting it. Hence, BETA can detect quantitative differences in TF activity between NDV and IAV responses. The estimation of full PDFs by BETA has significant advantages over single P-values. P values reflect the certainty of differential-activity, not its strength, and are sensitive to both the number of differentially-expressed genes, as well as the overall number of genes measured. BETA is robust to the number of differentially expressed genes in different experiments, while maintaining sensitivity to response-specific gene expression changes. BETA also allows comparison between different platforms by using a normalization factor based on the marginal probability of the number of differentially expressed genes. Other methods, such as QuSAGE [41] can also quantify gene set activity, but we found that BETA has better power for detecting transcription factor activity (Additional file 1: Text S3). BETA can also be used to quantify the activity of gene sets based on pathways or functional relationships, and details of comparison of BETA with other methods have been provided in the Additional file 1: Text S3. Thus, BETA should aid future studies seeking to identify differences between the responses to different IAV strains (e.g., comparing seasonal and pandemic strains), and can be employed with other gene set definitions (e.g., pathways from Reactome [42, 43], or functional groups from the Gene Ontology [44]).

Genome-wide transcriptional profiles provide a global view of host responses. However, it is important to remember that such measurements are population averages, and can be greatly impacted by heterogeneity. Although our experiments use a single cell type, virus infection itself produces two major subpopulations with unique responses. Virally-infected cells (expected to be ~63 % of the population at an MOI of one) activate pathways associated with virus recognition and up-regulate interferon. Interferon induces an anti-viral state in non-infected cells restricting spread of the virus and resulting in significant transcriptional changes in non-infected cells [45]. Other cytokines such as TNF released by infected cells contribute to these transcriptional changes through paracrine signaling. Infected cells can also respond to these cytokines, but their response may be modulated by viral antagonism. Our results show how this population heterogeneity can potentially mask important effects. ISG activity was detected following both infection with IAV and NDV. From this simple observation, one might conclude that the IAV strain of IAV does not effectively antagonize the interferon response. However, comparison with the NDV response using BETA revealed that the interferon response was significantly reduced following IAV infection. Using a combination of mathematical modeling and experiments at different MOIs, we demonstrated that the observed expression of ISGs is mainly coming from non-infected cells. Thus, the IAV strain does effectively antagonize the interferon response, but specifically in infected cells. While future experiments might be designed to separately profile infected and non-infected cells, these can be difficult to recognize [46].

The ability of BETA to quantitatively compare gene set activities was critical for carrying out a comparative analysis of transcription factor activities following IAV and NDV infection. This analysis revealed several effects of IAV immune antagonism, including the previously identified transcription factor NFATC4, which is associated with the inflammatory response during symptomatic IAV infections [11]. Additional effects predicted by BETA should be followed-up with detailed mechanistic studies. For example, IAV infection was predicted to suppress SATB1 activity in dendritic cells. Significantly, SATB1 was recently shown to be induced downstream of IRF7 in DCs [47]. Here, we experimentally confirmed that IAV infection modulates SATB1 nuclear translocation and DNA binding to target genes. The role of SATB1 in thymocytes and Th2 cells has been described, and it will be interesting to see if this factor has a similar function in DCs. Depending on the adaptor with which SATB1 co-operates it can have a role as an inducer or a suppressor of gene expression. For instance, SATB1 induces c-Maf [48]. During NDV infections of dendritic cells SATB1 induces expression of its putative target genes, such as PRDM1. This expression is suppressed during IAV infections; which is presumably due to reduced binding of SATB1 as observed during ChIP-PCR experiments. Interestingly, higher nuclear translocation of SATB1 observed during IAV infections suggests that IAV inhibits binding of SATB1 to the promoter. This is consistent with the role of SATB1 as a molecular adaptor for several other proteins that work to pack DNA into an inactive state. Thus, by addressing the computational problem of comparative analysis of transcription factor activities, we identified novel effects of IAV mediated antagonism.

Similar to other enrichment methods, such as QuSAGE [41] and GSEA [27], BETA is dependent on the quality of gene set definitions. Frequently, a large overlap between gene-sets makes it difficult to identify the true causal pathway or TF. However, combining enrichment results with other relevant information, such as TF mRNA levels, fold changes and location of the binding site (e.g., distance to the transcription start site), facilitate the selection of the most promising candidates for follow-up studies. Gene-sets could also include false positives; this is especially likely for computationally identified TF targets, which are known to have low specificity. Nevertheless, we find that BETA performs well in practice compared to other over-representation analysis and functional scoring methods (Additional file 1: Text S3). The statistical model underlying BETA is based on the binomial distribution, and thus assumes a fixed probability that each differentially expressed gene is a transcription factor target. For this reason, BETA should not be used for small gene set sizes.

## Conclusions

We have developed a computational framework (BETA) that enables quantitative comparative analysis of transcription factor activities. The source code is freely available at clip.med.yale.edu/beta. This method will aid future studies to identify mechanistic differences in the host-pathogen interactions. Application of BETA to genome-wide transcriptional profiling data, from human DCs identified SATB1 as a novel effect of IAV antagonism.

## Materials and methods

### Differentiation of DCs

All human research protocols for this work were reviewed and approved by the IRB of the Mount Sinai School of Medicine. Monocyte-derived DCs were obtained from healthy anonymous human blood donations obtained from New York Blood Center, following a standard protocol described elsewhere [49]. No information on age, sex or ethnicity from these donors is available. These donations are considered scientific samples and not human subjects according to the IRB guidelines. Briefly, human peripheral blood mononuclear cells (PBMCs) were isolated from buffy coats by Ficoll density gradient centrifugation and positive immunomagnetic purification for CD14 followed by a 5 day incubation with 500 U/ml hGM-CSF (Preprotech, Rocky Hill NJ) and 1000 U/ml hIL-4 (Preprotech, Rocky Hill NJ) All experiments were replicated using cells obtained from different donors.

### IFNB stimulation microarray experiments

Total RNA was purified from 2.5 × 10^6^ DCs per time sample using RNeasyMicro (Qiagen, Valencia, CA) with DNase treatment. RNA was eluted from columns using water and quantified by spectrophotometry. Quality control of RNAs was performed using the Agilent Bioanalyzer (Agilent, Santa Clara, CA). Total RNA from cells treated with 2000U/ml IFNB or control were harvested at 2.5 h. Naive DCs served as negative control. Three biological replicates were performed and RNA was reverse transcribed using T7-oligo(dT)24 to yield double-stranded cDNA. cRNA was transcribed and biotinylated from cDNA templates. cRNA was hybridized at 58C for 14 h to Affymetrix HU133plus2 Gene Chip Arrays (Affymetrix, Santa Clara, CA) by the Mount Sinai Microarray Shared Resource Facility. Details can be found at http://support.illumina.com/content/dam/illumina-support/documents/myillumina/3466bf71-78bd-4842-8bfc-393a45d11874/wggex_direct_hybridization_assay_guide_11322355_a.pdf*.* All microarrays studied in this paper have been deposited in the Gene Expression Omnibus (www.ncbi.nlm.nih.gov/geo) with the series accession number GSE54970.

### IAV and NDV expression microarray used to identify mechanisms of IAV antagonoism

Raw expression data of IAV and NDV infections was obtained from our previous work (GEO IDs GSE41067 and GSE18791, for IAV and NDV, respectively) [13, 14]. Briefly, pellets of monocyte derived dendritic cells were resuspended with IAV (New Caledonia/20/1999) and NDV stocks at multiplicity of infection of 1 and 0.5, respectively. RNA was collected at seven common time points: pre-infection and 2, 4, 6, 8, 10, and 12 h post-infection. The IAV samples were hybridized to HumanHT-12 v4 Expression BeadChip Kit (Illumina San Diego, CA) and NDV samples were hybridized to Affymetrix HU133plus2 Gene Chip Arrays (Affymetrix, Santa Clara, CA). Illumina arrays were log-transformed and quantile normalized by using Lumi package. Affymetrix arrays were normalized using GCRMA from simpleaffy package. Differential expression was defined for probes at each infection time-point using three criteria: (1) a minimum expression intensity of 7 or 5 for at least one time point for the Illumina and Affymetrix platforms, respectively, (2) an absolute fold-change of at least two relative to the pre-infection time-point, (3) a significant change in expression by LIMMA (BioConductor implementation) after correction for multiple hypothesis testing by false discovery rate (q < 0.05). Previously described criterias were used for the background set of genes [13, 14]. All analysis was performed using the BioConductor software package in R [50].

### Transcription factor target identification

Using the UCSC Genome Bioinformatics site, we downloaded the transcription start site data (TSS) for all human RefSeq genes, defined by the January 2010 refGene table [51]. The region +/-2Kb around each TSS was identified within a genome-wide multiple alignment of 45 vertebrate species to the human genome [52], also available through the UCSC Genome Bioinformatics site. In order to identify putative transcription factor binding sites, the human sequences, along with aligned regions from mouse, were masked for repetitive elements using RepeatMasker [53] and then analyzed using the TRANSFAC MATCH [54] algorithm with a cutoff, as defined within the database, chosen to minimize the sum of false positives and false negatives. The analysis was performed for all high quality vertebrate transcription factor matrices in the 2011.1 release of TRANSFAC [55], and putative binding sites were considered to be evolutionarily conserved if matches were also found at the aligned positions in the mouse sequences and had no gaps present in the multiple alignment between the species being compared. Each TRANSFAC matrix was linked to a set of gene symbols describing potential binding factors using annotations present in the “Binding Factor” field of the database. Only vertebrate TRANSFAC matrices that could be linked to a HGNC gene symbol, either directly or through an alias listed in NCBI gene, were included.

### Virus preparation and viral infection

The Newcastle disease virus (rNDV/B1) was generated in Prof. Peter Palese’s laboratory [21]. Influenza A/New Caledonia/20/1999, (H1N1) was obtained from Prof. Adolfo Garcia-Sastre’s laboratory [56]. For infection, virus stocks were diluted in serum free medium and pelleted DCs were resuspended into it at a multiplicity of infection of 0.5, 1 and 2. DCs from donors were infected in triplicate with the influenza strain A/New Caledonia/20/1999 or Newcastle disease virus for 10 min in RPMI medium at 37 °C. After infection cells were centrifuged to remove the viral inoculation media, and resuspended in culture medium. Infectivity of cells were measured after 8 h by NP staining. Cells were fixed with paraformaldehyde, permebealized with methanol, washed thrice with staining buffer and then stained with antibody against NP for 2 h. IFNA, MX1 and IP10 expression was measured by PCR. Samples were fixed with 1 % (PFA) at 0 (control), 2, 4, 6, 8 and 10 h post-infection for translocation and CHIP-PCR assays.

### Assessment of SATB1 translocation

Permeabilized infected DCs were stained with monoclonal antibodies for the transcription factor SATB1 (Abcam) and a nuclear stain Hoechst 33342 (Sigma). Cells were imaged with the imaging flow cytometer (Amnis). Pearson correlation coefficient was computed over the masked portion of image pixel intensities of the nuclear stain and the transcription factor. Nuclear translocation was assessed using the log2 transformed and control-normalized value of the computed Pearson correlation coefficient.

### Chromatin Immunoprecipitation (ChIP)-PCR

DNA bound proteins were cross linked to DNA by exposing cells to 1 % PFA for 10 min at RT. Cross linking was quenched with 0.25 M glycine. Cell nuclei were extracted and lysed with 3 different buffers used in a sequential order. Buffers contained 50 mM HEPES-KOH, 140 mM NaC, 1 mM EDTA, 10 % glycerol, 0.5 % NP-40, 0.25 % Triton X-100 (Buffer 1); 200 mM NaCl, 1 mM EDTA 0.5 mM EGTA, 10 mM Tris pH 8.0 (Buffer 2); 1 mM EDTA, 0.5 mM EGTA, 10 mM Tris pH 8.0, 100 mM NaCl, 0.1 % Na-Deoxylcholate and 2.5 mL N-lauroyl sarcosines. Chromatin was sheared 8 times for 30 s with 30 s breaks at 4C in Buffer 3 with a CHIP grade sonicator (Diagenode). Chromatin immunoprecipitation and subsequental DNA extraction was performed with the Auto Transcription ChIP kit and Auto IPure kit using the SX-8G IP-Star® Compact Automated System (all Diagenode) following the manufacturers protocol. The extent of SATB1 bound DNA was measured by PCR comparing the eluate of the chromatin immunoprecipitated sample to its corresponding input sample.

### Model development

To better understand how the observed gene expression patterns could be affected by underlying population heterogeneity, we developed a dynamic model of ISG induction. The model describes expression kinetics of two genes, G_i_ and G_u_, that are expressed by infected and non-infected cells respectively as:

1 $$ \frac{d{G}_i}{dt}\kern0.5em =\kern0.5em {k}_i\left(1\kern0.5em \hbox{-} \kern0.5em {\mathrm{e}}^{\hbox{-} \mathrm{M}\mathrm{O}\mathrm{I}}\right)\frac{B^n}{B^n+{H}^n}\kern0.5em -\kern0.5em {d}_i{G}_i $$

2 $$ \frac{d{G}_u}{dt}={k}_u{\mathrm{e}}^{\operatorname{}\hbox{-} \mathrm{M}\mathrm{O}\mathrm{I}}\frac{B^n}{B^n+{H}^n}-{d}_u{G}_u $$

where MOI indicates the multiplicity of infection used for dendritic cell infections, *k*_*i*_ and *k*_*u*_ are the rates of expression of genes G_i_ and G_u_, respectively, while *d*_*i*_ and *d*_*u*_ are the rates at which gene *G*_*i*_ and *G*_*u*_ are degraded, respectively. B is the level of Interferon-β, whose values are interpolated from the normalized IFNB1 gene expression levels, and is used as a surrogate for virus detection by the cell and *H* is a hill constant describing concentration of B when the rate of gene induction is half of its maximum rate. (Additional file 2: Figure S1).

### Numerical simulations and parameter estimation

Numerical simulations and parameter estimation were performed in Matlab R2010b. Ode45 was used to numerically simulate the ordinary differential equation models with zero initial condition for eight hours. For comparison with the simulated expression levels, experimentally measured gene expression levels, including IFNB1, were normalized by the maximum of the three averages calculated for each set of triplicate measurements at three multiplicities of infections. Experimentally measured IFNB1 values were used as input to the simulation, and values at non-measured time-points were obtained using linear interpolation (interp1) of the average normalized IFNB1 levels at each MOI.

Parameter estimation was performed for each gene separately. All the parameters (k, d, n and H) for a given gene were estimated simultaneously by defining an objective function which minimizes the difference between simulated and experimentally-observed expression levels at three MOIs. Both global and local search algorithms were used (Matlab code is provided in the Additional file 1: Text S4). The differential evolution algorithm (devec3) was run for 200 iterations and initial parameter values k = 0.0-2,n = 1.0-10,d = 0-1,H = 0-1 [57]. The resulting best estimates were then refined using a local search algorithm, non-linear least-squares (lsqnonlin), using the same objective function [58, 59]. The quality of parameter estimates was assessed by simulating the data with a set of randomly chosen parameters and analyzing its recall.

### Availability of supporting data

The microarray data used in this study are available in the GEO repository: GSE41067, GSE18791 and GSE54970. Source code for BETA is available at http://clip.med.yale.edu/beta.

## Additional files

Additional file 1:
**Details about methodology and comparison with other methods. Text 1.** Estimating transcription factor activities with BETA, **Text 2.** Transcription factors identified by BETA and differentially expressed at the mrna level. **Text 3.** Comparative analysis of BETA with existing methods. **Text 4.** Matlab code for parameter estimation of simulated data. Additional file 2:
**Figures showing normalized levels of IFNB1 (Figure S1) and identifiability of model parameters using the proposed estimation protocol (Figure S2).**

## Abbreviations

BETA: Bayesian Estimation of Transcription factor Activity
NC: New Caledonia
NDV: Newcastle Disease Virus
MOI: Multiplicity of Infection
IAV: Influenza A Virus
PFA: Paraformaldehyde
NP: Nucleoprotein
