# Supplementary material for: Comparative analysis of anti-viral transcriptomics reveals novel effects of influenza immune antagonism
Source: BMC Immunol. 2015 Aug 14;16:46. doi: 10.1186/s12865-015-0107-y (PMC4536893; doi:10.1186/s12865-015-0107-y)
Supplement: Additional file 1: — Details about methodology and comparison with other methods. Text 1. Estimating transcription factor activities with BETA, Text 2. Transcription factors identified by BETA and differentially expressed at the mrna level. Text 3. Comparative analysis of BETA with existing methods. Text 4. Matlab code for parameter estimation of simulated data. [file 12865_2015_107_MOESM1_ESM.docx]

**Supplementary text 1: Estimating transcription factor activities with BETA**

The observed frequency of TF targets ($\pi$) and the activity A are characterized by a full probability density function (PDF) that is estimated using a Bayesian approach [^1^](#_ENREF_1). Briefly, given the observed number of differentially-expressed TF target genes (x), we estimate:

$$P(\pi|x)=\frac{P(x|\pi)P(\pi)}{P(x)}=\frac{\left(\begin{matrix} N \\ x \end{matrix}\right)\pi^{x}(1-\pi)^{N-x}\beta(\pi|a,b)}{P(x)}$$

where P(x|π) is a Binomial likelihood function that accounts for total number of differentially-expressed genes (N). P(π) is a prior, which is modeled here as a β distribution (β(π|a,b)), as it forms a conjugate pair with the Binomial likelihood (i.e., using a β prior implies a β posterior). Finally, P(x) is the marginal probability of x and serves as a normalization factor. The hyperparameters for the Bayesian prior (a and b) are estimated as previously described [^1^](#_ENREF_1).

The probability density function of A is estimated by the following transformation.

$$P\left( A|x \right)dA=P\left( \pi| x \right)\left| \frac{d\pi}{dA} \right|d\pi=\frac{\left( 1-\hat{\pi} \right)\hat{\pi}e^{A}}{\left( 1-\hat{\pi} \right)^{2}+{2\hat{\pi}\left( 1-\hat{\pi} \right)e^{A}+\hat{\pi}}^{2}e^{2A}}P(\pi(A)|x)d\pi$$

Where $\pi=\frac{\frac{\hat{\pi}}{1-\hat{\pi}}e^{A}}{1+\frac{\hat{\pi}}{1-\hat{\pi}}e^{A}}$, the parameter $\hat{\pi}$ is a point estimate which is calculated as the proportion of TF targets among the set of background genes and $P\left( \pi\left( A \right) | x \right)d\pi$is the *β* posterior PDF from following equation: $P(\pi|x)=\frac{P(x|\pi)P(\pi)}{P(x)}=\frac{\left(\begin{matrix} N \\ x \end{matrix}\right)\pi^{x}(1-\pi)^{N-x}\beta(\pi|a,b)}{P(x)}$

In this way, we arrive at a full PDF for the TF activity A.

**Supplementary text 2: Transcription factors identified by BETA and differentially expressed at the mRNA level**

1. IRF8
2. IRF9
3. IRF7
4. IRF1
5. STAT1
6. POU2F1
7. TCFAP4
8. POU2F1
9. MYOD1
10. NKX6-2
11. NEUROD1

**Supplementary text 3: Comparative analysis of BETA with existing methods.**


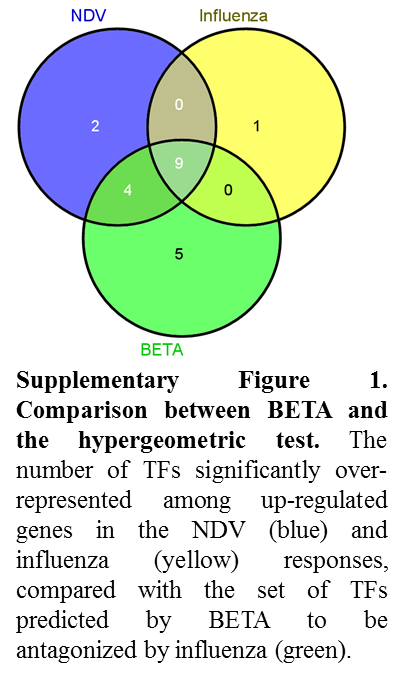
**Comparison with the hypergeometric test**: A hypergeometric test was carried out independently on the influenza and NDV data to identify individual transcription factors whose targets were over-represented among differentially expressed genes. Each time-point was analyzed separately using the formula in ^[2](#_ENREF_2)^. Set intersections were used to identify the overlap between TFs with significant activity in the influenza and NDV responses (Supplementary Figure 1). Effects of influenza-mediated antagonism were defined as TF with predicted activity in the NDV response, but not in the influenza response. By this definition, the hypergeometric test predicted 6 TFs whose activity was modulated by influenza. Four of these TFs (67%) were also predicted by BETA. The remaining two are NR1H4 and DMRTA2. The BETA p-value for DMRTA2 was 0.06, just missing the cutoff for significance. In addition, BETA detected significant differences in 9 TFs that were identified as active in both the influenza and NDV responses by the hypergeometric test. In these case, BETA revealed a quantitative suppression of activity that was not detectable by the hypergeometric test. In particular, IRF9, a well-known target of immune antagonism by the influenza NS1 protein, was not detected by the hypergeometiric test, but was revealed by BETA. This prediction of quantitative suppression was validated in the main text.


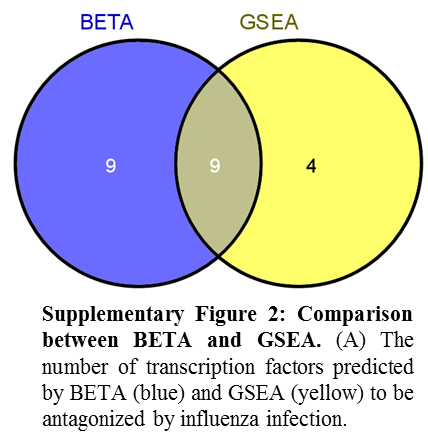
**Comparison with gene-set enrichment analysis (GSEA)**[^3^](#_ENREF_3): The GSEA was set up to identify the transcription factors differentially expressed between NDV and influenza. Specifically, GSEA was run using the gene-sets for each TRANSFAC binding site (see methods). Since the typical GSEA analysis is suited for gene-expression changes in control and treatment groups performed in one batch it could not be directly used for different experiments. Hence, we ranked the genes based on the difference between the NDV and influenza fold-changes obtained at each time-point upon infection with respect to control. GSEA predicts 13 TFs that are modulated by influenza infection. Nine (69%) of these are also identified by BETA (Supplementary Figure 2), including IRF9, which was missed by the hypergeometric test. The 4 TF predicted by GSEA, but not BETA, are MTF1, ZFP384, ZNF384, MEF2A, DMRTA2. BETA detects 9 TFs that are not identified by GSEA. This includes SATB1 which was experimentally validated to be antagonized by influenza. A significant disadvantage of GSEA is the difficulty of making post-hoc comparisons, since the calculated enrichment scores cannot be statistically compared. In contrast, BETA activity PDFs allow for post-hoc analysis. It should also be noted that BETA and GSEA are fundamentally different approaches. BETA in an over-representation analysis, while GSEA is one of the functional class scoring methods, which each have pros and cons as discussed in ^[4](#_ENREF_4)^.

**
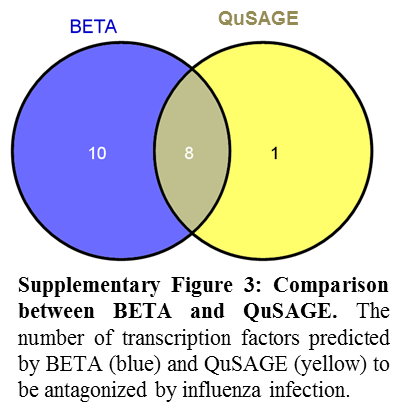
** **Comparison with QuSAGE^[5](#_ENREF_5)^:** QuSAGE predicts 9 TFs that are differentially modulated by influenza infection compared with NDV. Eight of these are also identified by BETA (Supplementary Figure 3), including IRF9 and SATB1. Only one TF (ARNT) was predicted by QuSAGE, but not BETA. However, ARNT activity is also not predicted by GSEA or the hypergeometric test. Similar to BETA, QuSAGE quantifies the activity rather than detecting it, but has less power compared with BETA. Based on our previous experience with QuSAGE, we suspect this is due to the very large gene set sizes when analyzing transcription factor targets.

**Supplementary text 4: Matlab code for parameter estimation of simulated data.** This code was run with Matlab version MATLAB R20120b. The devec3 function downloaded from http://www1.icsi.berkeley.edu/~storn/code.html.

*#Define variables*

global MOI; #multiplicity of infection used in the experiment

global MOI_list; #List of all multiplicities of infections for which the experiments are done

global out; #List of optimized parameters

global genData;#matrix to store simulated data

MOI_list=[2.0,1.0,0.5]; #List of all multiplicities of infections for which the experiments are done

all_data=zeros(3,8);#Matrix to store the data

tspan = [0:0.01:8.0]; # Time points

numparam=4;# number of parameters

inparameters=zeros(50,numparam);#List of input parameters

outparameters=zeros(50,numparam); #List of output parameters

for gen=1:50# for loop to generate a data-set

genData=zeros(3,length(tspan));# matrix to store simulated data

while(1)

ki=random('Uniform',0.0,2.0);#randomly choose a parameter

n=random('Uniform',1.0,10.0); #randomly choose a parameter

d=random('Uniform',0.0,1.0); #randomly choose a parameter

k=random('Uniform',0.0,1.0); #randomly choose a parameter

inparameters(gen,:)= [ki,n,d, k];#Store randomly chose parameters

for m = 1:length(MOI_list)#Generate the simulated data using inparameters

MOI=MOI_list(m);

U=(exp(-MOI_list(m)));#non-infected cells

I=(1-exp(-MOI_list(m)));#infected cells

y0 = zeros(1);#input for ode45

[T,Y]=ode45(@derivs,tspan,y0,options1, [ki,n,d, k]); #Simulate the data

genData(m,:)=Y;#simulated data

end #end of for

end #end of while

gloout=devec3('globalresiduals',VTR, D, [0.0,1.0,0.0,0.0],[2.0,10.0,1.0,1.0],all_data,D,200,0.1);#global

optimization by using devec3 algorithm to find the parameters using simulated data

out = lsqnonlin(@residuals,globalout,[0.0,0.0,0.0,0.0],[2.0,10.0,1.0,1.0]); ]); #local optimization using

least-square method and parameters from devec3 as input

outparameters(gen,:)=out; #recall of the parameters

end #end of the for loop.

**REFERENCES**

1. Yaari G, Uduman M, Kleinstein SH. Quantifying selection in high-throughput Immunoglobulin sequencing data sets. Nucleic acids research;40:e134.

2. Zaslavsky E, Hershberg U, Seto J, et al. Antiviral response dictated by choreographed cascade of transcription factors. J Immunol 2010;184:2908-17.

3. Subramanian A, Tamayo P, Mootha VK, et al. Gene set enrichment analysis: a knowledge-based approach for interpreting genome-wide expression profiles. Proceedings of the National Academy of Sciences of the United States of America 2005;102:15545-50.

4. Khatri P, Sirota M, Butte AJ. Ten years of pathway analysis: current approaches and outstanding challenges. PLoS computational biology 2012;8:e1002375.

5. Yaari G, Bolen CR, Thakar J, Kleinstein SH. Quantitative set analysis for gene expression: a method to quantify gene set differential expression including gene-gene correlations. Nucleic acids research 2013;41:e170.
