# Supplementary material for: Comparative analysis of anti-viral transcriptomics reveals novel effects of influenza immune antagonism
Source: BMC Immunol. 2015 Aug 14;16:46. doi: 10.1186/s12865-015-0107-y (PMC4536893; doi:10.1186/s12865-015-0107-y)
Supplement: Additional file 2: — Figures showing normalized levels of IFNB1 (Figure S1) and identifiability of model parameters using the proposed estimation protocol (Figure S2). [file 12865_2015_107_MOESM2_ESM.pdf]

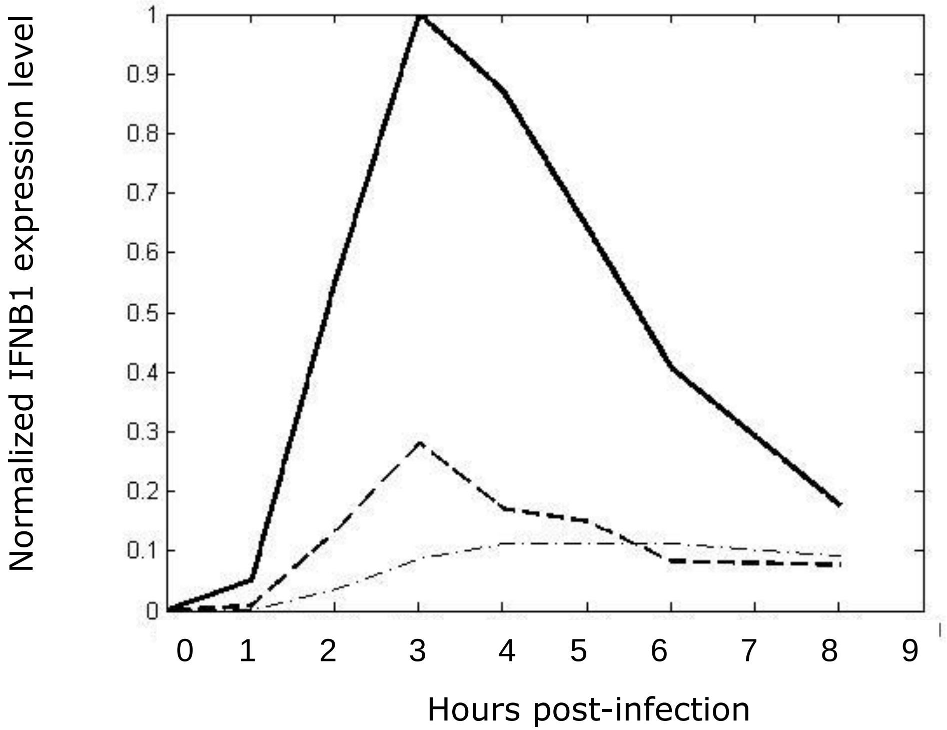

Supplementary Figure 1: Normalized IFNB1 expression time course used in the ODE model. IFNB1 mRNA levels were normalized by their highest value within each replicate. Values between measures points were determined by linear interpolation of their experimentally measured values at a MOI of 2 (thick solid line), 1 (thick dashed line) and 0.5 (think dashed dot line).

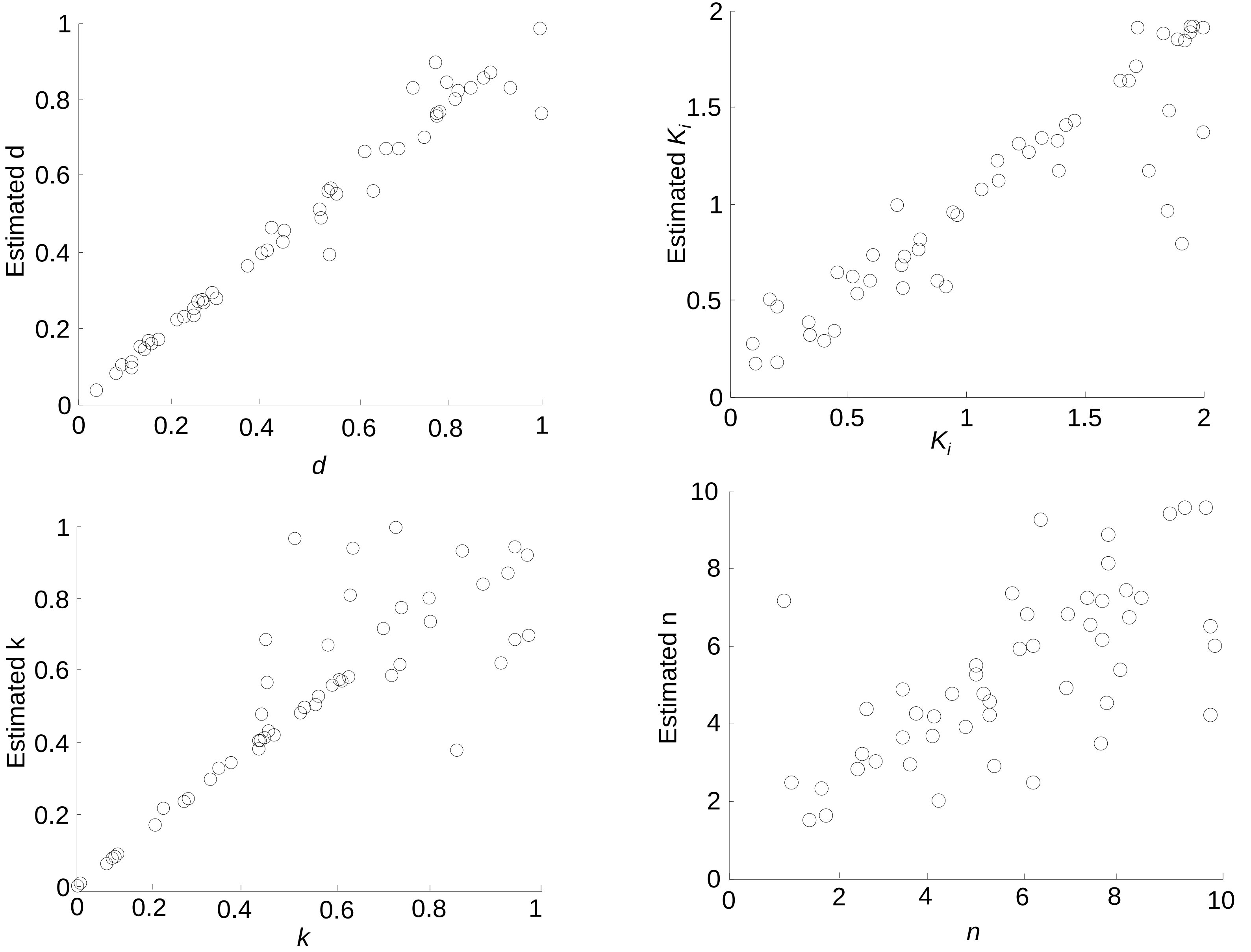

Supplementary Figure 2: Identifiability of model parameters using the proposed estimation protocol. As described in methods, simulated data sets were generated using a range of values for (A) degradation rate (*d*), (B) rate of expression of gene by infected cells (k_i), (C) the level of IFNB1 for which the gene expression rate is 0.5 per cell (*k*), and (D) Hill coefficient (*n*). Parameters were then estimated from these data and compared to their actual values.
